# *Tyrosinase-Cre*-Mediated Deletion of the Autophagy Gene *Atg7* Leads to Accumulation of the RPE65 Variant M450 in the Retinal Pigment Epithelium of C57BL/6 Mice

**DOI:** 10.1371/journal.pone.0161640

**Published:** 2016-08-18

**Authors:** Supawadee Sukseree, Ying-Ting Chen, Maria Laggner, Florian Gruber, Valérie Petit, Ionela-Mariana Nagelreiter, Veronika Mlitz, Heidemarie Rossiter, Andreas Pollreisz, Ursula Schmidt-Erfurth, Lionel Larue, Erwin Tschachler, Leopold Eckhart

**Affiliations:** 1 Research Division of Biology and Pathobiology of the Skin, Department of Dermatology, Medical University of Vienna, Vienna, Austria; 2 Department of Ophthalmology, Medical University of Vienna, Vienna, Austria; 3 Christian Doppler Laboratory for Biotechnology of Skin Aging, Vienna, Austria; 4 Institut Curie, PSL Research University, INSERM U1021, CNRS UMR3347, Normal and Pathological Development of Melanocytes, Orsay, France; 5 INSERM, Orsay, France; 6 Equipe labellisée – Ligue Nationale contre le Cancer, Université Paris 11, Orsay, France; Indiana University School of Medicine, UNITED STATES

## Abstract

Targeted gene knockout mouse models have helped to identify roles of autophagy in many tissues. Here, we investigated the retinal pigment epithelium (RPE) of *Atg7*^*f/f*^ *Tyr-Cre* mice (on a C57BL/6 background), in which Cre recombinase is expressed under the control of the tyrosinase promoter to delete the autophagy gene *Atg7*. In line with pigment cell-directed blockade of autophagy, the RPE and the melanocytes of the choroid showed strong accumulation of the autophagy adaptor and substrate, sequestosome 1 (Sqstm1)/p62, relative to the levels in control mice. Immunofluorescence and Western blot analysis demonstrated that the RPE, but not the choroid melanocytes, of *Atg7*^*f/f*^ *Tyr-Cre* mice also had strongly increased levels of retinoid isomerohydrolase RPE65, a pivotal enzyme for the maintenance of visual perception. In contrast to *Sqstm1*, genes involved in retinal regeneration, i.e. *Lrat*, *Rdh5*, *Rgr*, and *Rpe65*, were expressed at higher mRNA levels. Sequencing of the *Rpe65* gene showed that *Atg7*^*f/f*^ and *Atg7*^*f/f*^ *Tyr-Cre* mice carry a point mutation (L450M) that is characteristic for the C57BL/6 mouse strain and reportedly causes enhanced degradation of the RPE65 protein by an as-yet unknown mechanism. These results suggest that the increased abundance of RPE65 M450 in the RPE of *Atg7*^*f/f*^ *Tyr-Cre* mice is, at least partly, mediated by upregulation of *Rpe65* transcription; however, our data are also compatible with the hypothesis that the RPE65 M450 protein is degraded by *Atg7*-dependent autophagy in *Atg7*^*f/f*^ mice. Further studies in mice of different genetic backgrounds are necessary to determine the relative contributions of these mechanisms.

## Introduction

Autophagy is an intracellular process that delivers organelles as well as proteins and other molecules to the lysosomes for degradation by hydrolytic enzymes [1–4]. Macroautophagy, the main type of autophagy, comprises the enclosure of substrates by a vesicle, the autophagosome, and the subsequent fusion of the vesicle with a lysosome. Genetic disruption of essential autophagy-related genes (*Atg*s) such as *Atg7* suppresses autophagy and allows *in vivo* studies to unravel new roles of autophagy. Recently, we have generated *Atg7*^*f/f*^ *Tyr-Cre* mice [5], in which the exon encoding the catalytic domain of Atg7 is flanked by loxP sites [6] and Cre recombinase is expressed under the control of the tyrosinase promoter [7]. In these mice, Atg7 is inactivated specifically in pigment cells such as the melanocytes of the skin [5].

The retinal pigment epithelium (RPE) is a monolayered epithelium that supports the visual function and survival of retinal photoreceptor cells. In vertebrates, vision is initiated in rod and cone photoreceptors. The photosensitive entity of the visual pigment rhodopsin in photoreceptor cells consists of opsin and the 11-*cis*-retinal chromophore. Upon absorption of light by the pigments, 11-*cis*-retinal is isomerized to all-*trans*-retinal, which leads to conformational changes of opsin and subsequent visual phototransduction. All-*trans*-retinal is then transported from photoreceptors to RPE cells, where regeneration of 11-*cis*-retinal occurs through a molecular cascade known as visual cycle. An essential step of the visual cycle, isomerization of all-*trans*- retinyl ester to 11-*cis*-retinol is catalysed by retinal pigment epithelium-specific 65 kDa protein (RPE65) [8,9]. Lecithin-retinol acyltransferase (LRAT) and retinol dehydrogenase 5 (RDH5) catalyze the reactions before and after the RPE65-mediated step of the visual cycle, respectively. Clinically, mutations in RPE65 are associated with type II Leber’s congenital amaurosis (LCA2), a hereditary retinal blinding disease caused by RPE65 deficiency [10]. Correction of RPE65 deficiency in patients with LCA2 by gene therapy improves vision by re-activating the retinoid cycle [11].

Autophagy has been reported to counteract disease-associated processes and to support the normal function of the RPE. In particular, autophagy has been suggested to prevent or delay age-related macular degeneration by removing cytotoxic protein aggregates and oxidation products in the RPE [12–17]. Moreover, autophagy is impaired by lysosomal dysfunction in a mouse model of juvenile neuronal ceroid lipofuscinosis (Batten disease) [18] and in mice lacking βA3/A1-crystallin in the RPE, resulting in pathological signs similar to those of patients with age-related macular degeneration [19]. Targeted gene deletions were also used to determine functions of individual components of molecular machinery of autophagy in the RPE. The lack of the autophagy regulator *Rb1cc1* was shown to induce degeneration of the RPE in the mouse [20]. The RPE-specific deletion of the autophagy gene *Atg5* diminished the phagocytosis and degradation of photoreceptor outer segments [21], suggesting that components of the molecular machinery of autophagy are involved in the maintenance of normal vision. Recently, *Atg7* was deleted in an inducible and RPE-specific manner using mice that carry *Atg7* flanked by loxP sites, *Cre* under the control of the tetracycline-responsive element (TRE) and a transgenic reverse tetracycline-dependent transactivator (rtTA) gene driven by the RPE-specific human vitelliform macular dystrophy-2 (*VMD2*) promoter [22]. In contrast to RPE lacking Atg5 [21], deletion of Atg7 did not reduce levels of 11-*cis*-retinal [22], indicating differences in the functions of Atg5 and Atg7 in non-canonical autophagy-related processes and/or effects of the genetic background of the different mouse models [22].

Here we investigated the impact of Tyr-Cre-mediated abrogation of Atg7-dependent autophagy on the RPE of *Atg7*^*f/f*^ *Tyr-Cre* mice on a C57BL/6 background [5]. We report that this mouse model of autophagy deficiency in pigment cells displays elevated expression of RPE65 and other key enzymes of the visual cycle in the RPE.

## Materials and Methods

### Mice and RPE cell isolation

*Atg7*^*f/f*^ mice were kindly provided by Masaaki Komatsu (Tokyo Metropolitan Institute of Medical Science, Tokyo, Japan). The use of the *Tyr-Cre* transgene for conditional gene deletion in the RPE has been reported recently [23]. The generation, genotyping and maintenance of *Tyr-Cre* mice, *Atg7*-floxed mice, and *Atg7*^*f/f*^ *Tyr-Cre* mice have been described previously [5–7]. Comparisons were made between age-matched (at least 10 months old) mice of the two genotypes. Hemizygous males and homozygous females were used whereas heterozygous females were excluded to avoid possible effects of X chromosome inactivation on the *Tyr-Cre* transgene which is located on that chromosome [24].

For histological investigations, the eyes were enucleated and fixed in 4% paraformaldehyde. Subsequently, the eyes were embedded in paraffin and thin-sections were stained with hematoxylin & eosin (H&E) or subjected to immunolabelling as described below. For RNA and protein analyses of the RPE, the eyes were enucleated and incubated with dispase II from *Bacillus polymyxa* (Roche, Basel, Switzerland) (10 mg/ml) in DMEM with 10% fetal calf serum. After overnight enzymatic digestion at 4°C, an incision was made at the ora serrata and extended circumferentially through the whole eye globe. After removing the anterior segment, vitreous and neuroretina, four radial incisions were placed in the posterior segment to divide it into 4 tissue strips. Finally, RPE cells were peeled off as an intact sheet from the underlying choroidoscleral tissue in each strip by Trouman-Barraquer corneal microforceps (Accutome, The Netherlands) under a dissecting microscope.

### Ethics statement

Mice were bred and sacrificed by cervical dislocation for the preparation of tissue samples according to the animal welfare guidelines of the Medical University of Vienna, Austria, as approved by the Ethics Review Committee for Animal Experimentation of the Medical University of Vienna, Austria and the Federal Ministry of Science, Research and Economy, Austria (Zl. 1712/115-1997/98-2013). No experiments on live animals were performed.

### Immunofluorescence analysis

Immunofluorescence analysis was performed according to a published protocol [25] with modifications. The sections were incubated with polyclonal rabbit anti-Sqstm1/p62 (MBL International Corporation, dilution 1:1000) and monoclonal mouse anti-RPE65 (Abcam, dilution 1:500) followed by incubations with goat anti-rabbit and goat anti-mouse immunoglobulin antibodies conjugated to Alexa-Fluor 546 and Alexa-Fluor 488 (Molecular Probes, Leiden, The Netherlands), respectively, for 30 minutes. Hoechst 33258 (Molecular Probes) was used to label nuclear DNA. Appropriate isotype antibodies were used for negative controls. The labelled sections were photographed under a fluorescence microscope using the Metamorph software.

### Western blot analysis

RPE cell sheets were lysed in a protein extraction buffer containing 50 mM Tris (pH 7.4), 2% SDS and complete protease inhibitor cocktail (Roche, Mannheim, Germany) and homogenized by sonication. The insoluble debris was removed by centrifugation and the protein concentration of the supernatant was measured by the bicinchoninic acid (BCA) method (Pierce, Rockford, IL). Western blot analysis was performed as described previously [26]. 20 μg protein was loaded per lane on SDS polyacrylamide gels (ExcelGel SDS, gradient 8–18, Amersham Biosciences) on a horizontal electrophoresis system (Amersham Biosciences). After gel electrophoresis and blotting of proteins onto a nitrocellulose membrane using the Multiphor II Electrophoresis system (Amersham Biosciences), the proteins were visualized by staining with Ponceau dye. For the detection of specific antigens, the following first step antibodies were used: rabbit polyclonal anti-p62 (BML-PW9860-0100, Enzo Life Sciences, NY, dilution 1:2000), rabbit polyclonal anti-LC3 (GTX82986, GeneTex, Irvine, CA, dilution 1:2000), and mouse monoclonal anti-RPE65 (ab13826, Abcam, Cambridge, MA, dilution 1:2500). As secondary antibodies, goat anti-rabbit immunoglobulin G (IgG) (Bio-Rad Laboratories, CA) and sheep anti-mouse IgG (NA931V, GE Healthcare Limited, UK) antibodies conjugated to horseradish peroxidase were used at a dilution of 1:10000. The bands were revealed with enhanced chemiluminescence reagent (ThermoFisher Scientific).

### RNA preparation, reverse transcription and quantitative PCRs

Total RNA was prepared from surgically isolated RPE sheets using the RNeasy Plus Mini kit (Qiagen, Hilden, Germany) according to the manufacturer’s instructions. RNA was reverse transcribed using the iScript cDNA synthesis kit (Bio-Rad Laboratories, Hercules, CA) according to the manufacturer’s protocol. TaqMan gene expression assays were performed on an ABI Prism 7500 Fast Real-Time PCR System (Perkin Elmer, Applied Biosystems) with incubations at 50°C for 2 minutes followed by denaturation at 95°C for 10 minutes and 45 cycles of 95°C for 15 seconds and 60°C for 1 minute. The following Taqman Gene expression assays containing specific primers and 6-carboxyfluorescein (FAM)-conjugated probes were purchased from Life Technologies (Carlsbad, CA): *Gapdh* (cat# Mm99999915_g1), *Sqstm1*/*p62* (cat# Mm00500417_m1), *Rpe65* (cat# Mm00504133_m1). The “delta-delta Ct method” was used for quantifications and averaged relative quantification (RQ) values ± standard deviation (SD) normalized to the expression level of glyceraldehyde-3-phosphate dehydrogenase (*Gapdh*) were calculated. Furthermore, mRNAs of visual cycle regulators of the RPE were quantified by RT-PCR with SYBR-Green in the LightCycler system (Roche Applied Science, Mannheim, Germany) according to our previously established protocol [27]. Target gene expression was quantified using a mathematical model of Pfaffl [28]. The primers are listed in S1 Table. Statistical differences were assessed using the two-tailed t-test, with *p* <0.05 being considered significant.

### PCR amplification and DNA sequencing

DNA was isolated from tail tips of *Atg7*^*f/f*^ and *Atg7*^*f/f*^ *Tyr-Cre* mice (C57BL/6 background) as well as from a Krt2^+/-^ Krt10^-/-^ mouse (mixed C57BL/6 and BALB/c background [29]). Exon 13 of the Rpe65 gene was amplified with the primers Rpe65-s, 5´-ATAAAGCATCTTACTAACATCCA-3´ and Rpe65-a, 5´- CTTTCCTAATAGAGAACACACT-3´. Dream Taq polymerase (Thermo Fisher Scientific) was used in the following amplification program: 95°C for 2 minutes; 35 cycles of 95°C for 3 seconds, 60°C for 30 seconds, and 72°C for 17 seconds; 72°C for 10 minutes. The site corresponding to the Rd8 mutation in the *Crb1* gene was amplified with the primers Crb1-s, 5´-GGTGACCAATCTGTTGACAATCC-3´ and Crb1-a, 5´-GCCCCATTTGCACACTGATGAC-3´ according to a published protocol [30]. The PCR products were purified with the Wizard SV Gel and PCR clean-up system (Promega), and sequenced in both directions (Microsynth, Balgach, Switzerland).

### Statistics

The significance of differences between sample groups was examined using the two-tailed unpaired Student’s t-test. Differences were considered statistically significant when p < 0.05.

## Results

### Histological characterization of the RPE and choroid melanocytes in *Atg7*^*f/f*^ *Tyr-Cre* mice

**Fig 1:**
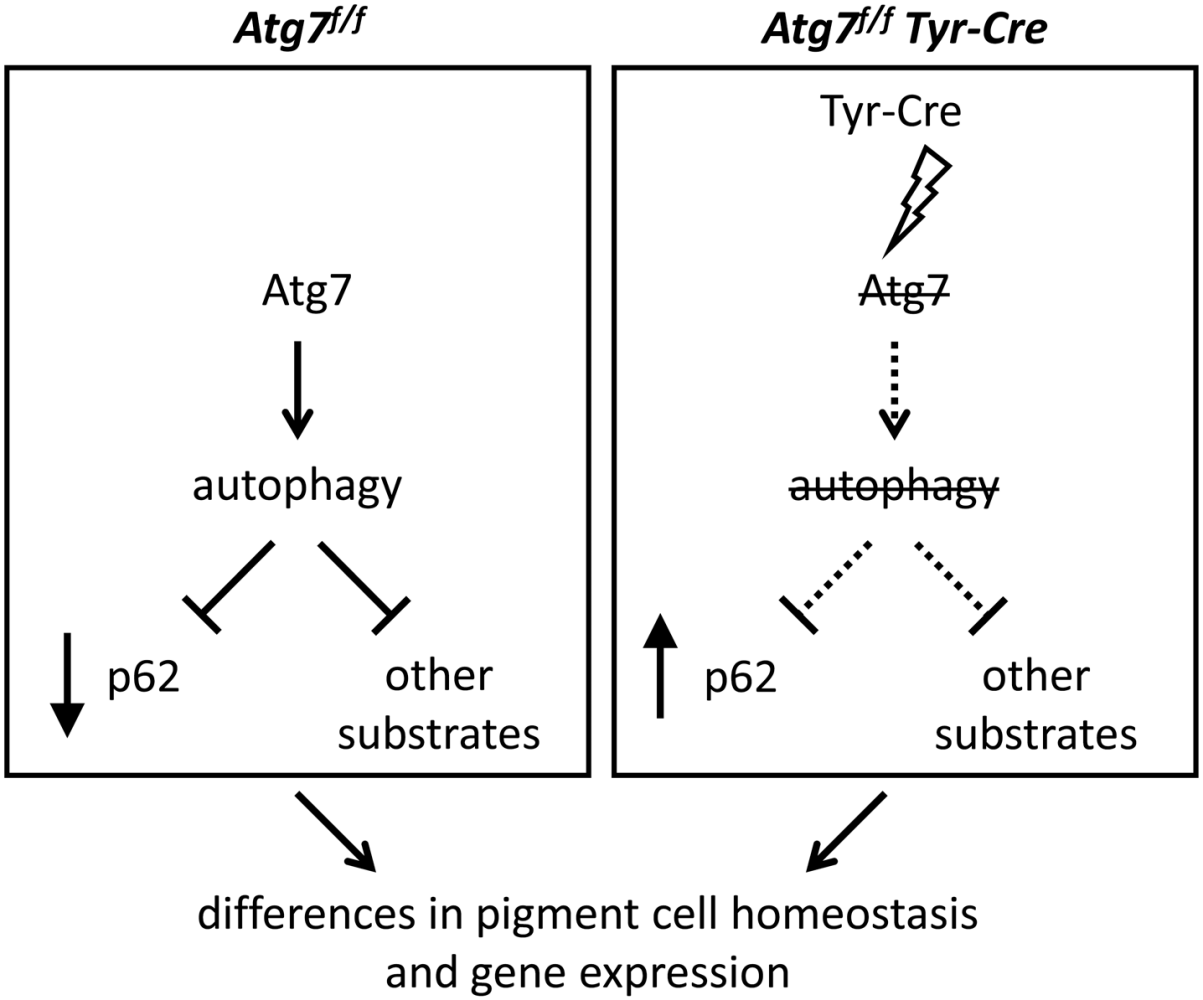
Hypothetical model of effects caused by *Tyr-Cre*-mediated deletion of Atg7. Expression of the Cre recombinase under the control of the tyrosinase promoter leads to deletion of the floxed *Atg7* gene, abrogation of autophagy and accumulation of p62 and other autophagy substrates in a pigment cell-specific manner. In addition to these direct effects, abrogation of autophagy alters cellular homeostasis that manifests in altered gene expression.

Previous investigations of *Atg7*^*f/f*^ *Tyr-Cre* mice have shown suppression of autophagy in skin melanocytes [5] while ocular pigment cells have remained uncharacterized in this mouse strain. Here we tested the hypothesis that RPE cells and choroid melanocytes of *Atg7*^*f/f*^ *Tyr-Cre* mice might be affected by the absence of autophagy, which can be evaluated by determining the abundance of the autophagy substrate protein Sqstm1/p62 [5], and by altered cellular homeostasis manifesting in altered gene expression (Fig 1).

Histological investigations showed that choroid melanocytes of *Atg7*^*f/f*^ *Tyr-Cre* mice were morphologically inconspicuous and normally pigmented (S1 Fig). This result is in line with our previous finding that *Atg7* is not essential for pigmentation and survival of cutaneous melanocytes *in vivo* [5]. Likewise, the RPE of *Atg7*^*f/f*^ *Tyr-Cre* mice was pigmented and showed no consistent differences to that of fully autophagy-competent *Atg7*^*f/f*^ control mice (S1 Fig). At the macroscopic level, the gross appearance of *Atg7*^*f/f*^ *Tyr-Cre* mouse eyes was normal, and mice up to an age of 2 years showed no signs suggestive of visual impairment.

### *Atg7*^*f/f*^ *Tyr-Cre* mice have increased expression of RPE65 at the mRNA and protein levels

**Fig 2:**
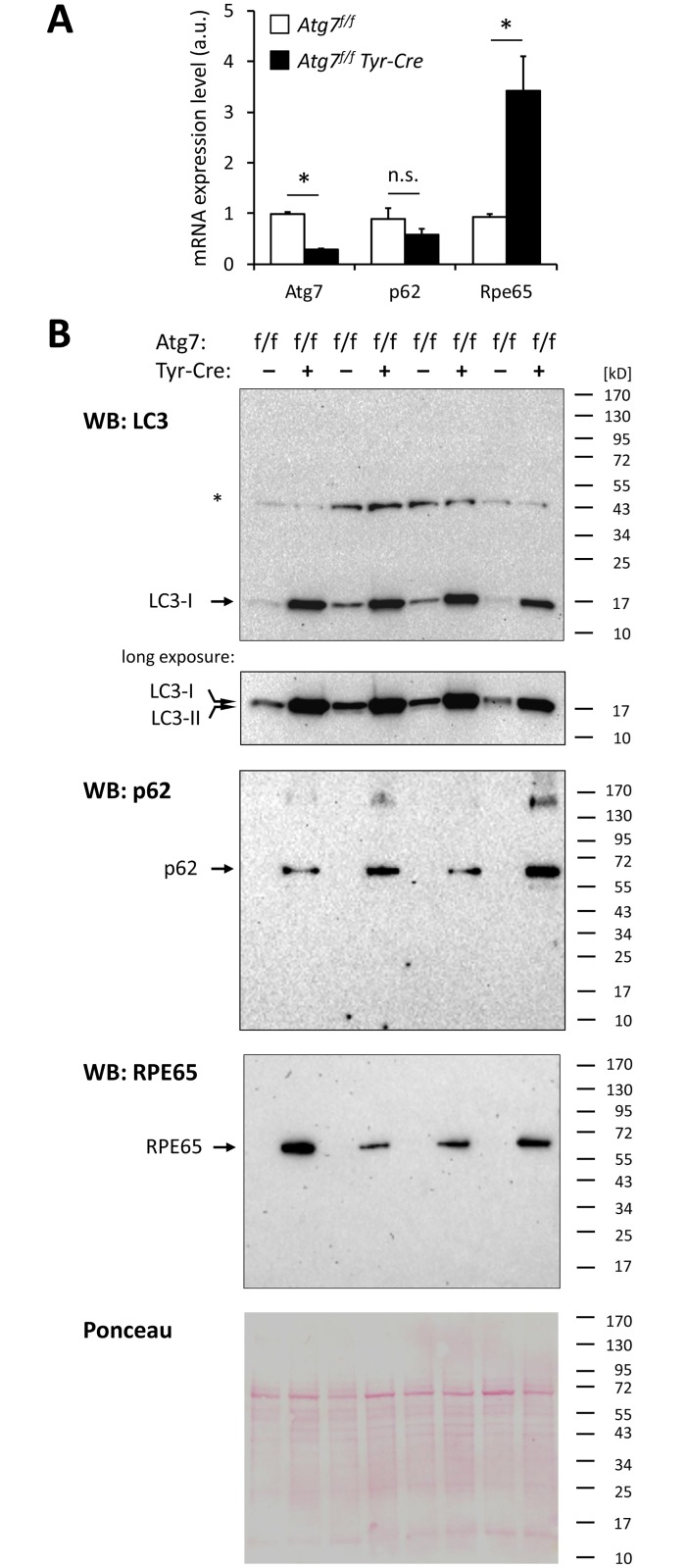
RPE65 but not p62 is transcriptionally upregulated in the RPE of *Atg7*^*f/f*^ *Tyr-Cre* mice. (A) RNAs from the RPE of *Atg7*^*f/f*^ and *Atg7*^*f/f*^ *Tyr-Cre* mice were analyzed by quantitative RT-PCR for Atg7, p62 and Rpe65. Expression levels (a.u., arbitrary units) are shown relative to the expression of the house-keeping gene *Gapdh*. n = 4 mice per genotype. Error bars indicate standard errors of the mean. *p<0.05, considered statistically significant (2-tailed t-test). n.s., not significant. (B) Western blot analysis of RPE lysates from *Atg7*^*f/f*^ and *Atg7*^*f/f*^ *Tyr-Cre* mice. Protein lysates obtained from freshly isolated RPE sheets of *Atg7*^*f/f*^ and *Atg7*^*f/f*^ *Tyr-Cre* mice were subjected to Western blot (WB) analysis for microtubule-associated protein light chain 3 (LC3), p62, and RPE65 (retinal pigment epithelium-specific 65 kDa protein). The same protein amounts were run on 3 separate electrophoresis gels. Before exposing the membrane to the primary antibodies, the membranes were stained with Ponceau reagent to visualize the total proteins on the membrane (loading control). A representative Ponceau staining confirming equal loading is shown at the bottom. Furthermore, the intensities of an unspecific band (*) show that there is not more protein loaded in *Atg7*^*f/f*^ *Tyr-Cre* than in *Atg7*^*f/f*^ lanes. A short and a long exposure of the LC3 Western blot are shown to demonstrate the accumulation of LC3-I, the non-lipidated form of LC3, in *Atg7*^*f/f*^ *Tyr-Cre* mice (short exposure), and weak bands corresponding to LC3-II, the lipidated form of LC3, in *Atg7*^*f/*f^ mice (long exposure). Note that under the Western blot conditions applied here the levels of p62 and RPE65 were below the detection threshold in RPE lysates from wild-type mice. Positions of protein size markers (kD, kilo-Dalton) are indicated on the right.

Quantitative RT-PCR analysis showed that Atg7 mRNA was present in the RPE and that it was strongly reduced in the RPE of *Atg7*^*f/f*^ *Tyr-Cre* versus RPE of *Atg7*^*f/f*^ control mice (Fig 2A). The transcriptional activity of the *Sqstm1/p62* gene (normalized to the expression of house-keeping gene *Gapdh*) was not different in RPE cells of *Atg7*^*f/f*^ *Tyr-Cre* and *Atg7*^*f/f*^ control mice. Unexpectedly, the expression of the *Rpe65*, which served as a marker gene of the RPE, was increased more than 3-fold in *Atg7*^*f/f*^ *Tyr-Cre* mice (*p*<0.05) (Fig 2A).

At the protein level, presence of Atg7 in the RPE of wild-type mice has previously been reported [22], however, in the present study the amounts of Atg7 in the RPE were below the detection limits of our well-established Western blot assay [31]. Because of this limitation, the downstream effects of *Atg7* deletion, that is, the accumulation of autophagy substrates, had to be used as indicator of suppressed autophagy in target cells. Western blot analysis of the essential autophagy adaptor and substrate, microtubule-associated protein light chain 3 (LC3) [32] showed that the non-lipidated isoform of LC3 (LC3-I) was strongly accumulated in the RPE of *Atg7*^*f/f*^ *Tyr-Cre* mice relative to that of *Atg7*^*f/f*^ (fully autophagy-competent) control mice (Fig 2B). Small amounts of lipidated (autophagosome-associated) LC3-II were detected in the RPE of *Atg7*^*f/f*^ mice upon long exposure of the membrane (Fig 2B). Another autophagy adaptor and substrate, Sqstm1/p62 was present at high amounts in the RPE of *Atg7*^*f/f*^ *Tyr-Cre* but undetectable at the protein level in the RPE of fully autophagy-competent *Atg7*^*f/f*^ mice (Fig 2). This marked accumulation of the p62 protein was in contrast to the equal abundance of p62 mRNA in the RPE of *Atg7*^*f/f*^ and *Atg7*^*f/f*^ *Tyr-Cre* mice, suggesting that p62 was expressed and efficiently degraded by autophagy in normal RPE and that this degradation was blocked when *Atg7* was deleted by the *Tyr-Cre* construct (Fig 2B).

In line with the transcriptional upregulation of *Rpe65* (Fig 2A), the RPE of *Atg7*^*f/f*^ *Tyr-Cre* mice contained elevated amounts of RPE65 protein (Fig 2B). The homeostatic levels of RPE65, that could be prepared from the eyes of *Atg7*^*f/f*^ control mice (on a C57BL/6 background), were below (Fig 2B) or just minimally above (S2 Fig) the threshold of detection of our assay. Ponceau staining of electrophoresed and membrane-transferred proteins showed that the sizes and abundance patterns of the cellular proteins of the RPE generally were not altered in *Atg7*^*f/f*^ *Tyr-Cre* mice, compared to the *Atg7*^*f/f*^ controls (Fig 2B, lowest panel).

**Fig 3:**
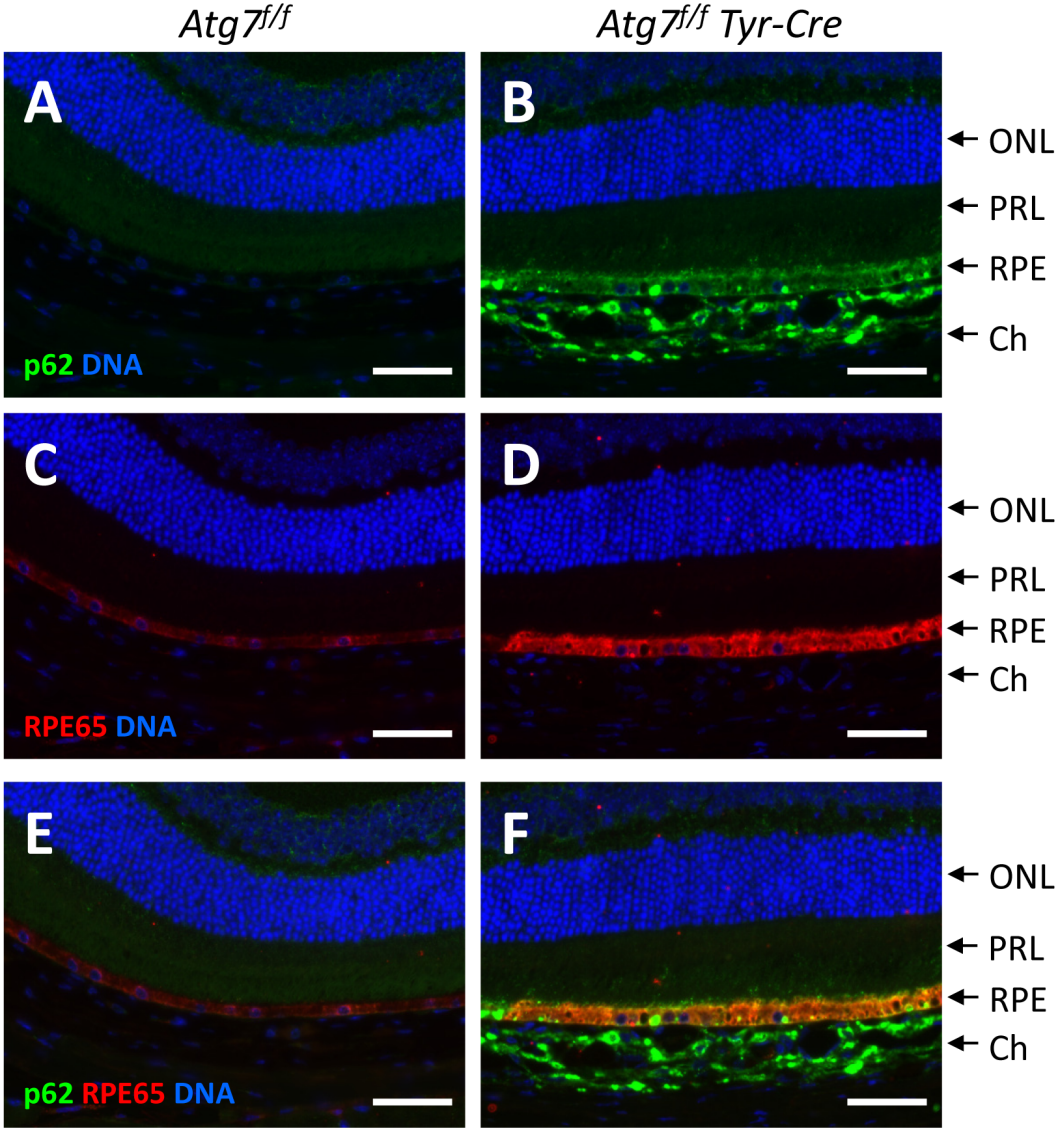
Deletion of Atg7 leads to the accumulation of p62 and RPE65 in the RPE. Double-immunofluorescence labelling of p62 (green) (A, B) and RPE65 (red) (C, D) in *Atg7*^*f/f*^ (A, C, E) and *Atg7*^*f/f*^ *Tyr-Cre* (B, D, F) eyes. Nuclear DNA was labelled with Hoechst 33258 (blue). Panels E and F show merged images. ONL, outer nuclear layer; PRL, photoreceptor layer; RPE, retinal pigment epithelium; Ch, choroid. Scale bars: 50 μm.

To identify the cells in which p62 and RPE65 accumulated *in situ*, we performed immunofluorescence analyses with anti-p62 and anti-RPE65 antibodies that gave bands of the expected sizes in Western blot analyses (Fig 2B). Sqstm1/p62 was highly abundant in both RPE cells and choroidal melanocytes of *Atg7*^*f/f*^ *Tyr-Cre* mice but absent in the equivalent cells of *Atg7*^*f/f*^ mice (Fig 3A and 3B). RPE65 was detected, as expected, specifically in the RPE of both *Atg7*^*f/f*^ and *Atg7*^*f/f*^ *Tyr-Cre* mice (Fig 3C–3F). In agreement with the Western blot results, the intensity of the RPE65 immunofluorescent signal was strongly increased in *Atg7*^*f/f*^ *Tyr-Cre* mice (Fig 3C and 3D; S3 Fig), indicating that the abundance of this key enzyme of the visual cycle was altered in the absence of autophagy. Immunofluorescence analysis of *Tyr-Cre* mice carrying the normal *Atg7* gene and wild-type mice on the same C57BL/6 background showed equal expression of RPE65 (S4 Fig), confirming that the expression of the Tyr-Cre gene alone did not cause alterations in RPE65 gene expression. Taken together, these results suggested that the accumulation of p62 protein in *Atg7*-deleted ocular pigment cells resulted from a defect in autophagic turnover in the absence of transcriptional upregulation, whereas the increased level of RPE65 protein in *Atg7*-deleted RPE cells was accompanied and possibly caused by an upregulation of *Rpe65* transcription via a yet-to-be-clarified mechanism.

Interestingly, previous studies have suggested that RPE65 is subjected to enhanced degradation in C57BL/6 mice as compared to other mouse strains [33–35]. This was attributed to a polymorphism of the *Rpe65* sequence that causes the mutation L450M in the C57BL/6 background, whereas *Rpe65* of BALB/c and 129/Ola mice encodes RPE65 L450 [33, 36]. Comparison of the RPE65 amino acid sequences showed that L450 is conserved in mammals and other vertebrates except for several reptilian species (S2 Table), indicating that L450 represents the ancestral amino acid residue in the mouse and M450 represents a mutation in the C57BL/6 line. We sequenced exon 13 of the *Rpe65* gene in *Atg7*^*f/f*^ and *Atg7*^*f/f*^ *Tyr-Cre* mice and found that the mice of our breeding colony were homozygous for M450 (S5 Fig). Thus, the results obtained in *Atg7*^*f/f*^ and *Atg7*^*f/f*^ *Tyr-Cre* mice are informative with regard to the RPE65 variant M450, whereas the effects of Tyr-Cre-mediated deletion of Atg7 on RPE65 L450, which corresponds to normal RPE65 in humans, remain to be determined in further studies.

### Regulators of the visual cycle are transcriptionally upregulated in *Atg7*^*f/f*^ *Tyr-Cre* mice

**Fig 4:**
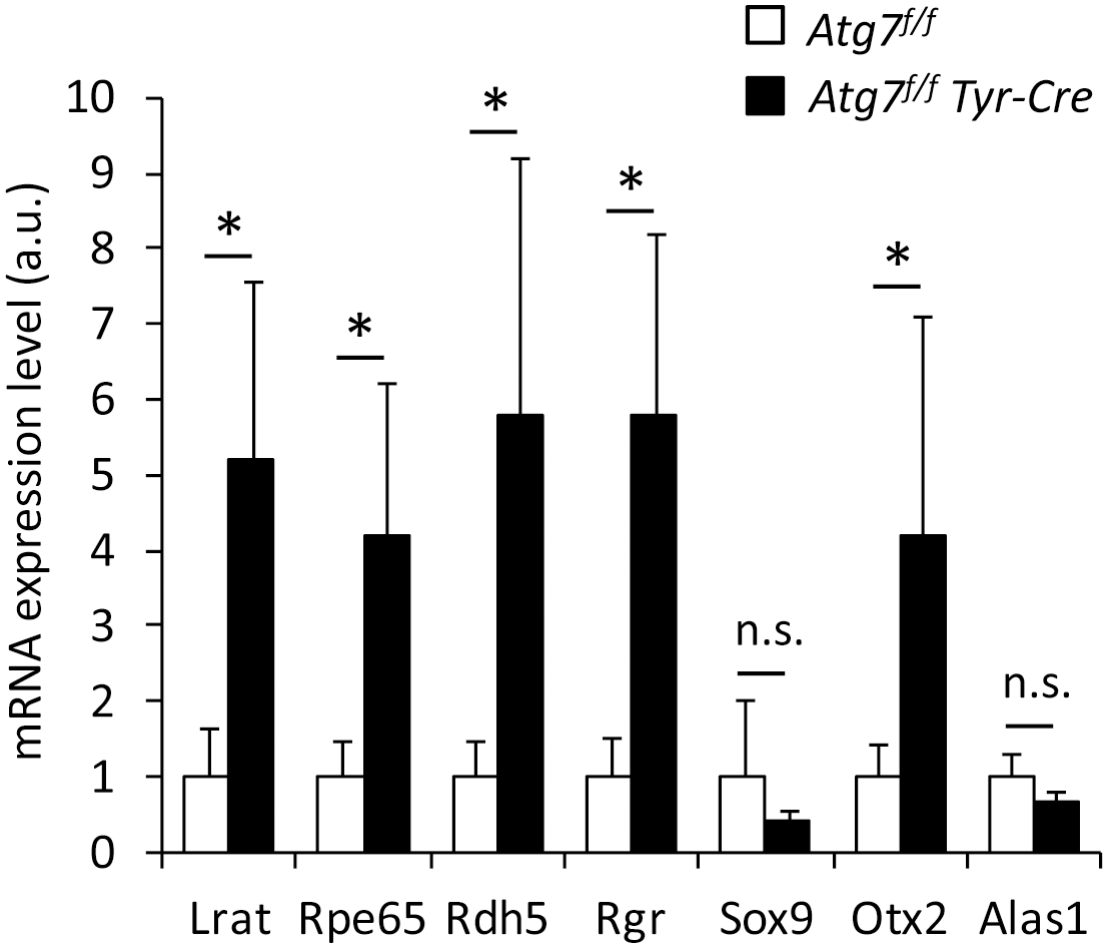
Regulators of the visual cycle are expressed at increased levels in the RPE of *Atg7*^*f/f*^ *Tyr-Cre* mice. The expression levels of genes encoding visual cycle regulators (*Lrat*, *Rpe65*, *Rdh5*, *Rgr*), transcription factors implicated in the homeostasis of the RPE (*Sox9*, *Otx2*), and house-keeping genes (*Alas1*, *B2m*) were determined by quantitative RT-PCR analysis of RNAs from the RPE of *Atg7*^*f/f*^ (n = 5) and *Atg7*^*f/f*^ *Tyr-Cre* (n = 4) mice. Expression levels (a.u., arbitrary units) are shown relative to the expression of the house-keeping gene *B2m*. Error bars indicate standard deviations. *p<0.05, considered statistically significant (two-tailed t-test). n.s., not significant.

As RPE65 was reported to be part of a transcriptionally regulated network of visual cycle proteins [37], we extended our gene expression analyses by quantitative RT-PCRs. Besides RPE65, the two other visual cycle enzymes of the RPE, i.e., LRAT and RDH5, as well as the crucial regulator protein, retinal G protein coupled receptor (RGR) [38], were expressed at significantly higher levels in the RPE of *Atg7*^*f/f*^ *Tyr-Cre* mice than in that of *Atg7*^*f/f*^ control mice (*p*<0.05) (Fig 4). We also investigated potential regulators of gene expression, i.e. SRY (sex determining region Y)-box 9 (SOX9) and the homeobox transcription factor orthodenticle homolog 2 (Otx2), two transcription factors implicated in the homeostasis of the RPE [37, 39]. The expression of SOX9 levels were not altered, whereas expression of Otx2 was significantly increased in the RPE of *Atg7*^*f/f*^ *Tyr-Cre* mice relative to the *Atg7*^*f/f*^ controls (Fig 4). Expression levels of the house-keeping gene *Alas1* were not significantly different in the RPE of both genotypes. Taken together, our results suggest that the upregulation of RPE65 in RPE cells of *Atg7*^*f/f*^ *Tyr-Cre* mice (on a C57BL/6 background) is accompanied by increased expression of other regulators of the visual cycle.

## Discussion

The results of this study establish *Atg7*^*f/f*^ *Tyr-Cre* mice as an animal model in which Atg7-dependent regulation of RPE65 expression can be investigated. *Atg7*^*f/f*^ *Tyr-Cre* mice were previously generated to determine the impact of autophagy suppression on melanocytes of the skin [5]. The results of the present study suggest that autophagy is also inhibited in choroidal melanocytes and in RPE cells of this conditional knockout mouse line. The accumulation of the autophagy substrate p62 in melanocytes of the choroid confirms the effectiveness of the *Tyr-Cre* transgene [7] to target melanocytes and supports our previous conclusion that *Atg7*-dependent autophagy is important for the homeostasis of melanocytes [5]. Importantly, p62 also accumulated in the RPE of *Atg7*^*f/f*^ *Tyr-Cre* mice, suggesting that the *Tyr* promoter-driven, Cre recombinase-mediated deletion of *Atg7* also takes place in RPE cells or in precursor cells during the development of the RPE.

Atg7 has been demonstrated to be expressed in RPE of wild-type mice both at the mRNA and protein level [22]. In the present study Atg7 mRNA was detected in RPE preparations and a significant reduction of Atg7 expression could be confirmed in the RPE of *Atg7*^*f/f*^ *Tyr-Cre* relative to *Atg7*^*f/f*^ mice. However, Atg7 protein could not be detected by Western blot analysis, most likely due to the limited sensitivity of our Atg7 Western blot protocol. The lack of a quantitatively confirmed reduction of Atg7 protein abundance in the RPE of *Atg7*^*f/f*^ *Tyr-Cre* mice limits the conclusions that can be drawn from the results of this study. However, the demonstration, by Western blot, of accumulation of LC3-I and p62 in the RPE of *Atg7*^*f/f*^ *Tyr-Cre* mice and the demonstration, by immunofluorescence, of accumulation of p62 in most cells of the RPE cells *in situ* (Fig 3B) suggest suppression of autophagy in the RPE cells of *Atg7*^*f/f*^ *Tyr-Cre* mice.

In contrast to other mouse lines in which autophagy was suppressed specifically in the RPE [21, 22], *Atg7*^*f/f*^ *Tyr-Cre* mice are supposed to have autophagy defects both in RPE cells and choroid melanocytes. Thus, this mouse model combines cell-intrinsic effects of autophagy suppression in the RPE and indirect effects that may depend on altered interactions of autophagy-deficient choroid cells with RPE cells. To dissect the relative contributions of these two types of effects of *Tyr-Cre*-mediated *Atg7* deletion in *Atg7*^*f/f*^ *Tyr-Cre* mice, comparisons with mice carrying gene deletions in single cell types will be necessary in the future.

Our results suggest that the RPE phenotype of *Atg7*^*f/f*^ *Tyr-Cre* mice differs from the phenotypes reported for other mouse models. RPE-specific deletion of the autophagy gene *Atg5* (on a C57BL/6 background) impaired a pivotal physiological function of RPE cells, *i*.*e*. LC3-associated phagocytosis of photoreceptor outer segments [21], although the general tissue organization of RPE remained undisturbed. By contrast, deletion of autophagy regulator *Rb1cc1* caused degeneration of the RPE [19]. Doxycycline-induced RPE-specific deletion of *Atg7* (on a BALB/c background) impaired neither RPE morphology nor retinoid recycling [22]. In the latter report, p62 was demonstrated to accumulate in the RPE whereas the expression levels of retinoid metabolizing proteins were not investigated. The results of the present study show that the RPE of *Atg7*^*f/f*^ *Tyr-Cre* mice (on a C57BL/6 background) was free from severe morphological abnormalities but contained massive aggregates of p62. It remains to be investigated whether the formation of p62-positive protein aggregates causes tissue damage or whether it actually protects RPE cells by sequestering potentially harmful substances [40, 41]. Of note, *Atg7*^*f/f*^ and *Atg7*^*f/f*^ *Tyr-Cre* mice do not contain the *Rd8* mutation of the *Crb1* gene, which has been reported to cause retinal degeneration in some commercially distributed C57BL/6 substrains [30, 42]. The phenotypic differences between mouse models carrying RPE-targeted deletions of autophagy-related genes likely reflect differences in the genetic backgrounds, differences in the employed gene deletion systems and differences in the functions of the target genes, with some, but not all, autophagy genes having roles outside of classical autophagy, such as *Atg5* in LC3-associated phagocytosis [21, 43]. In future studies it will be important to determine the retinoid content of the eyes, the kinetics of chromophore regeneration and parameters of retinal function in *Atg7*^*f/f*^ *Tyr-Cre* mice in comparison to other mouse models.

Our findings that, besides the characteristic changes in the abundance of the autophagy substrates p62 and LC3, Tyr-Cre-mediated deletion of Atg7 caused increased the abundance of RPE65 M450 and the expression of RPE-specific visual cycle genes, are surprising and potentially important. High levels of RPE65 were consistently detected *in situ* by immunofluorescence and in protein lysates using Western blot analysis as well as at the mRNA level using quantitative RT-PCRs in *Atg7*^*f/f*^ *Tyr-Cre* mice. Previous studies have demonstrated that the protein levels of RPE65 M450 are much lower than those of RPE65 L450 although the corresponding mRNAs are expressed at similar levels in the RPE of C57BL/6 and BALB/c mice, respectively [33]. This observation has led to the hypothesis that RPE65 M450 might be destabilized and degraded by an unknown mechanism [35]. Intriguingly, RPE65 M450 accumulates in the absence of Atg7-dependent autophagy. Therefore, it is tempting to speculate that RPE65 M450 is a substrate of autophagy in RPE cells carrying an intact *Atg7* gene (S6 Fig). Hypomorphic mutations (such as L450R) of human RPE65 are also associated with decreased RPE65 protein abundance and visual acuity [35, 44]. The interaction of autophagy with both normal and pathogenic RPE65 variant remains to be determined in future studies.

Besides the hypothetical and as-yet unproven direct autophagic degradation of RPE65 M450, increased transcription of the *Rpe65* gene is likely to contribute to the observed accumulation of RPE65 protein in *Atg7*^*f/f*^ *Tyr-Cre* mice. As the transcriptional upregulation of RPE65 occurred in concert with increased mRNA expression of at least 3 other regulators of the visual cycle, i.e., LRAT, RDH5 and RGR, all RPE-specific steps of the visual cycle appear to be augmented when RPE cells cope with metabolic changes induced by autophagy deficiency, at least in this mouse model. If the suppression of autophagy increases the abundance of a retinoid-metabolizing enzyme such as RPE65 M450, as in the speculative scenario outlined above (S6 Fig), altered concentrations of retinoid metabolites might influence the expression of genes involved in the visual cycle. In the context of this hypothesis, it is interesting that the administration of 9-cis-retinyl acetate, a prodrug for the generation of 9-cis-retinal, alters the expression levels of *Rpe65* and *Rgr* as well as that of the transcription factor *Otx2* in the eyes of C57BL/6 mice (see S1 Table in [45]).

The molecular mechanisms by which *Rpe65* and the genes encoding the other aforementioned proteins are transcriptionally upregulated in *Atg7*^*f/f*^ *Tyr-Cre* mice, remain to be determined. SOX9 may be a candidate transcription factor involved in the control of *LRAT*, *RDH5* and *RGR* expression [37], although its own expression level appeared to be unaffected by the suppression of autophagy in the RPE (Fig 4). Increased expression of Otx2, which has been reported to regulate the transcription of RPE65 [37], may contribute to the altered gene expression in the RPE of *Atg7*^*f/f*^ *Tyr-Cre* mice. Interestingly, RPE65 and Otx2 have been reported to be co-regulated by miR-410 [46]. Furthermore, the transcriptional upregulation of the aforementioned genes may be driven directly or indirectly by Nrf2, a transcription factor that is activated in the presence of high levels of p62 [39, 47] and contributes to the stress resistance of RPE cells [48–50]. Indeed, the transcription of known Nrf2 target genes such as *Gclc*, *Gstm1* and *Nqo1* was induced in the RPE of *Atg7*^*f/f*^ *Tyr-Cre* mice (not shown). Clearly, more studies are necessary to determine the mechanism of RPE65 gene regulation in *Atg7*^*f/f*^ *Tyr-Cre* mice. Considering the new concept of targeted alterations of gene expression for the therapy of diseases that affect the retina [51], studies of the *Atg7*^*f/f*^ *Tyr-Cre* mouse model may help to define strategies for modulating the metabolism of retinoids in the RPE.

In summary, the results of this study suggest that autophagy contributes to the control of the RPE65 variant M450 and that the complex regulatory network of the visual cycle responds to reduced levels of autophagy by increased expression of regulators of retinoid metabolism. Further investigations of *Atg7*^*f/f*^ *Tyr-Cre* mice and other models are necessary to explore whether and how these findings could open new therapeutic avenues for degenerative retinal diseases with impaired retinoid metabolism.

## Supporting Information

S1 Fig Histology of the retina and choroid of *Atg7*^*f/f*^ and *Atg7*^*f/f*^ *Tyr-Cre* mice. Hematoxylin and eosin (H&E) staining of retina and choroid from 3 *Atg7*^*f/f*^ (A, C, E) and 3 *Atg7*^*f/f*^ *Tyr-Cre* (B, D, F) mice at an age of at least 10 months. Size bars: 100 μm.
(PDF)

S2 Fig Western blot detection of RPE65 in the RPE of *Atg7*^*f/f*^ and *Atg7*^*f/f*^ *Tyr-Cre* mice. Protein lysates obtained from freshly isolated RPE sheets of *Atg7*^*f/f*^ and *Atg7*^*f/f*^ *Tyr-Cre* mice were subjected to Western blot (WB) analysis for RPE65 (retinal pigment epithelium-specific 65 kDa protein). Before exposing the membrane to the primary antibody, it was stained with Ponceau reagent to visualize the total proteins on the membrane (loading control). Note that in the Western blot only a faint band corresponding to RPE65 (arrow) was detected in RPE lysates from *Atg7*^*f/f*^ mice whereas the band was strong in RPE lysates from *Atg7*^*f/f*^ *Tyr-Cre* mice. In other experiments, the amount of RPE65 in *Atg7*^*f/f*^ samples was below the detection limit (Fig 2). Positions of protein size markers (kD, kilo-Dalton) are indicated on the right.
(PDF)

S3 Fig Immunofluorescence analysis of RPE65 in the retina of *Atg7*^*f/f*^ and *Atg7*^*f/f*^ *Tyr-Cre* mice. Immunofluorescence (IF) labelling of RPE65 (red) in *Atg7*^*f/f*^ (A, C, E) and *Atg7*^*f/f*^ *Tyr-Cre* (B, D, F) eyes. The mice were 10–12 months old. The results of 3 samples per genotype are shown (representative for >5 mice per genotype). Replacement of the anti-RPE65 antibody with an isotype control antibody abolished the specific labeling (G, H). Nuclear DNA was labelled with Hoechst 33258 (blue). The position of the RPE is indicated by a red arrow. Scale bars: 50 μm.
(PDF)

S4 Fig Immunofluorescence analysis of RPE65 in the retina of wild-type and *Tyr-Cre* mice. As a control experiment, sections through the eyes of wildtype (A, C) and *Tyr-Cre* (B, D) mice on a C57BL/6 background carrying the normal (not floxed) *Atg7* gene were immunofluorescence labelled for RPE65 (green). Nuclear DNA was labelled with Hoechst 33258 (blue). The results are representative for 3 mice per genotype. The position of the RPE is indicated by a green arrow. Scale bars: 20 μm.
(PDF)

S5 Fig Sequencing of *Rpe65* in *Atg7*^*f/f*^ and *Atg7*^*f/f*^ *Tyr-Cre* mice. Genomic DNA was prepared from *Atg7*^*f/f*^ (A) and *Atg7*^*f/f*^ *Tyr-Cre* (B) mice from the same breeding colony. DNA from an unrelated mouse on a mixed BALB/c and C57BL/6 background (C) was investigated for comparison. The DNAs were amplified with *Rpe65*-specific primers as described in the Material and Methods section, and sequenced. Representative sequence chromatograms are shown. The positions of the single nucleotide polymorphism leading to either M450 (codon: ATG) or L450 (codon: CTG) are indicated by arrowheads. The encoded amino acid residues and the numbers of the residues in the RPE65 protein are shown above the chromatograms.
(PDF)

S6 Fig Hypothetical model of interactions between autophagy, RPE65 M450 and gene expression in the RPE of mice on a C57BL/6 background. This model includes speculative elements besides changes observed in the study of *Atg7*^*f/f*^ and *Atg7*^*f/f*^ *Tyr-Cre* mice. According to this model, RPE65 M450 is largely degraded by autohpagy in RPE cells expressing Atg7 (*Atg7*^*f/f*^ (left panel)). It is possible that autophagy influences the expression of visual cycle genes (*Rpe65*, *Lrat*, *Rgr*) via unknown mechanisms (indicated by question marks). In *Atg7*^*f/f*^ *Tyr-Cre* RPE cells (right panel), RPE65 M450 is not degraded and reaches higher abundance. Possibly, the effect of RPE65 M450 on the retinoid metabolism or other mechanisms contribute to an upregulation of the transcripion of visual cycle genes including *Rpe65*. Importantly, several hypotheses depicted in this schematic remain to be tested in future studies.
(PDF)

S1 Table Primers for quantitative RT-PCRs using LightCycler technology. (PDF)

S2 Table Comparison of RPE65 amino acid (aa) sequences in vertebrates. (PDF)
