# Supplementary figures and images for: Tyrosinase-Cre-Mediated Deletion of the Autophagy Gene Atg7 Leads to Accumulation of the RPE65 Variant M450 in the Retinal Pigment Epithelium of C57BL/6 Mice
Source: PLoS One. 2016 Aug 18;11(8):e0161640. doi: 10.1371/journal.pone.0161640 (PMC4990303; doi:10.1371/journal.pone.0161640)

*Atg7<sup>f/f</sup>*

**A**

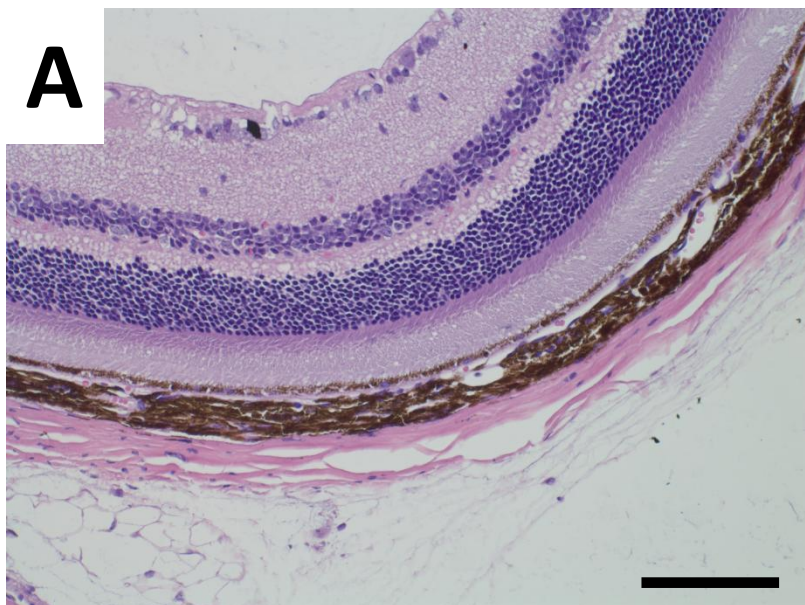

*Atg7<sup>f/f</sup> Tyr-Cre*

**B**

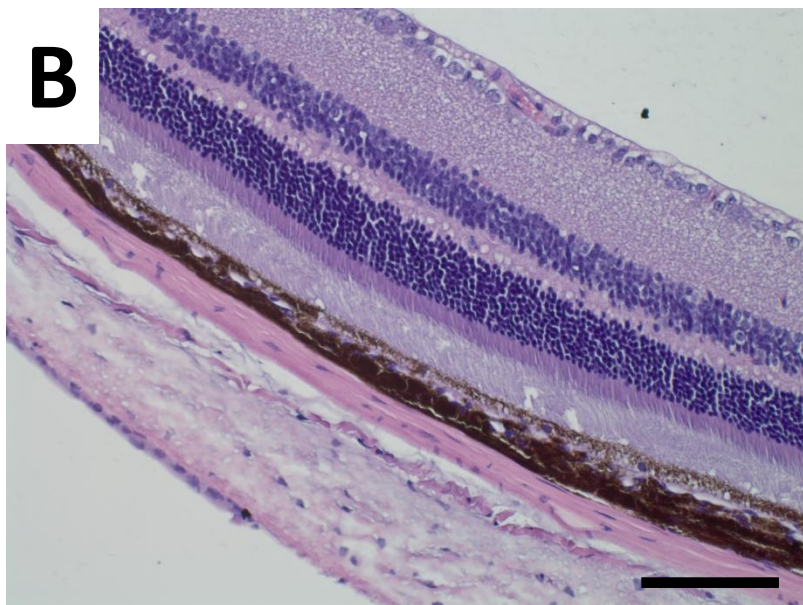

← GCL  
← IPL  
← INL  
← OPL  
← ONL  
← PRL  
← RPE  
← Ch

**C**

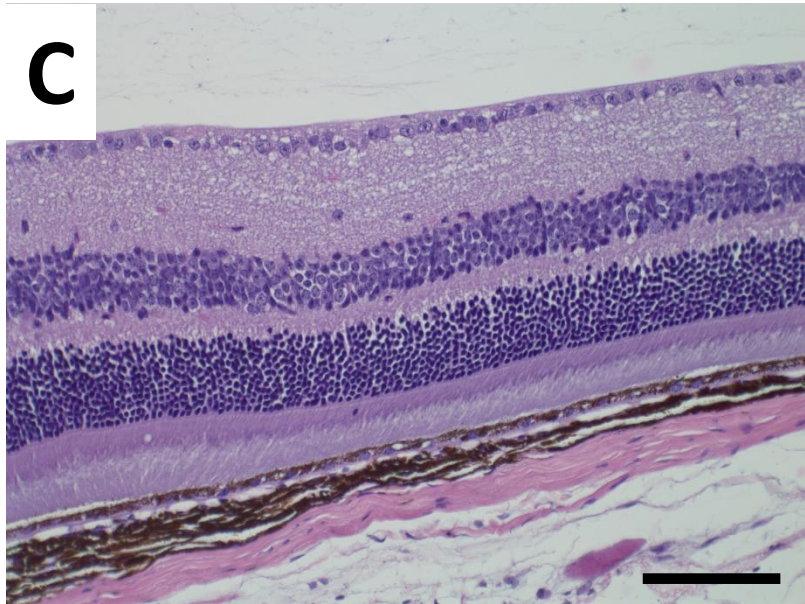

**D**

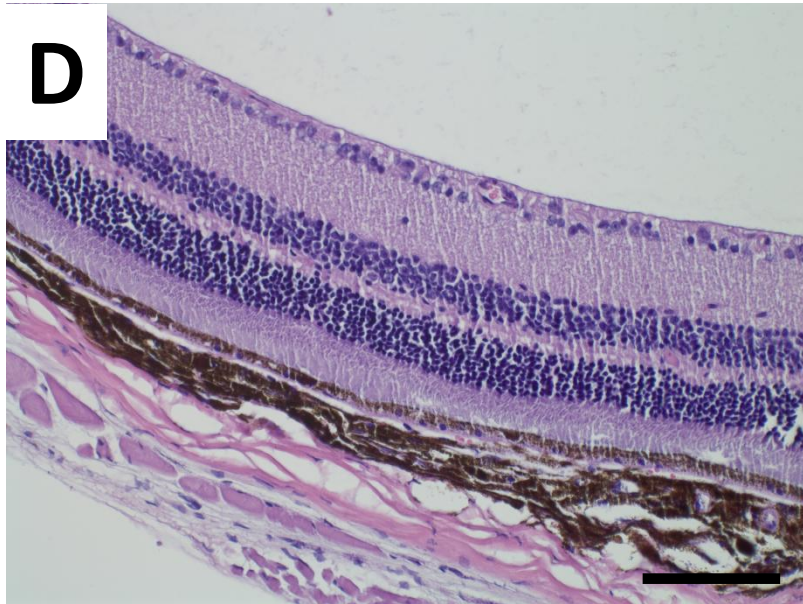

**E**

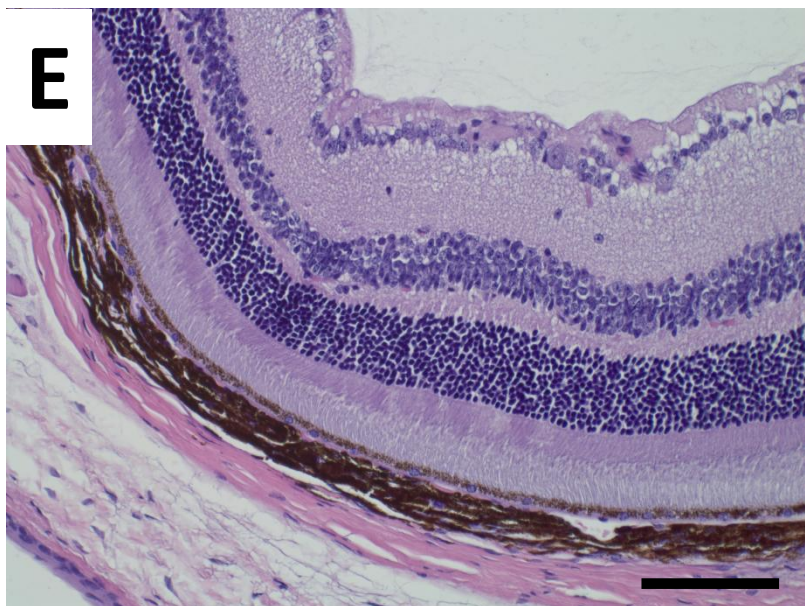

**F**

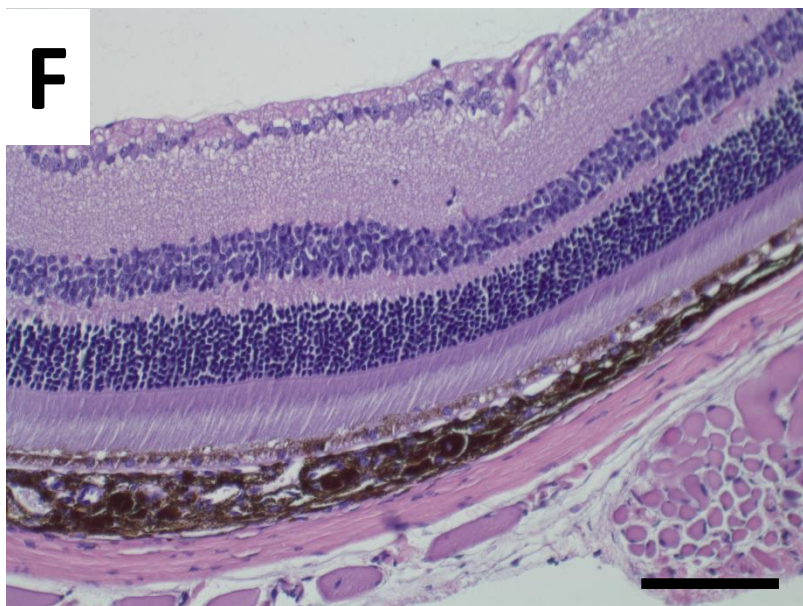

Supplement: S1 Fig — Hematoxylin and eosin (H&E) staining of retina and choroid from 3 Atg7f/f (A, C, E) and 3 Atg7f/f Tyr-Cre (B, D, F) mice at an age of at least 10 months. Size bars: 100 μm. (PDF) [file pone.0161640.s001.pdf]

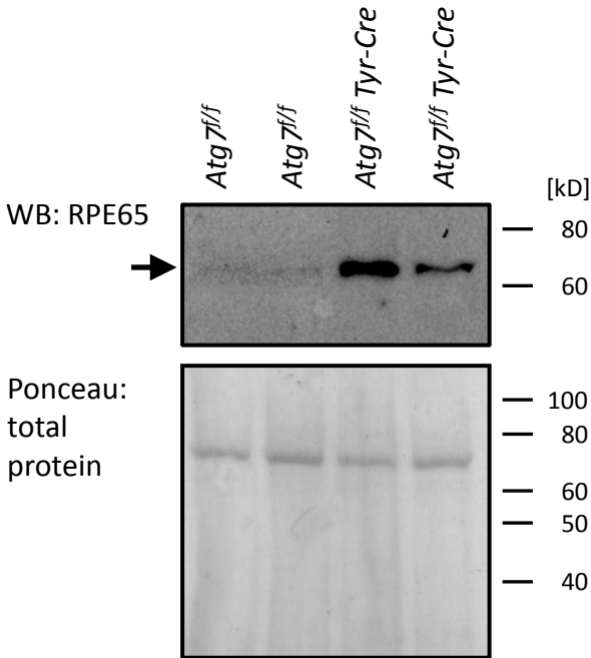

Supplement: S2 Fig — Protein lysates obtained from freshly isolated RPE sheets of Atg7f/f and Atg7f/f Tyr-Cre mice were subjected to Western blot (WB) analysis for RPE65 (retinal pigment epithelium-specific 65 kDa protein). Before exposing the membrane to the primary antibody, it was stained with Ponceau reagent to visualize the total proteins on the membrane (loading control). Note that in the Western blot only a faint band corresponding to RPE65 (arrow) was detected in RPE lysates from Atg7f/f mice whereas the band was strong in RPE lysates from Atg7f/f Tyr-Cre mice. In other experiments, the amount of RPE65 in Atg7f/f samples was below the detection limit (Fig 2). Positions of protein size markers (kD, kilo-Dalton) are indicated on the right. (PDF) [file pone.0161640.s002.pdf]

*Atg7<sup>f/f</sup>*

*Atg7<sup>f/f</sup> Tyr-Cre*

IF: RPE65

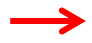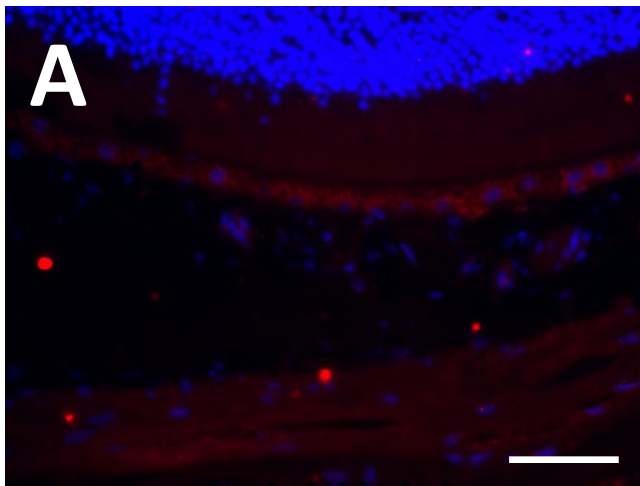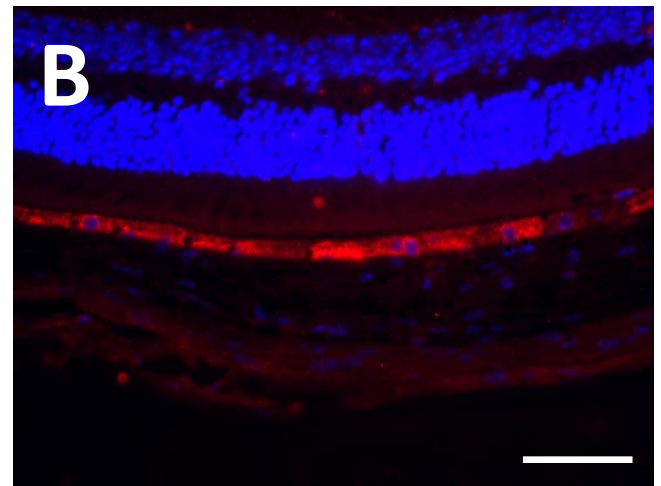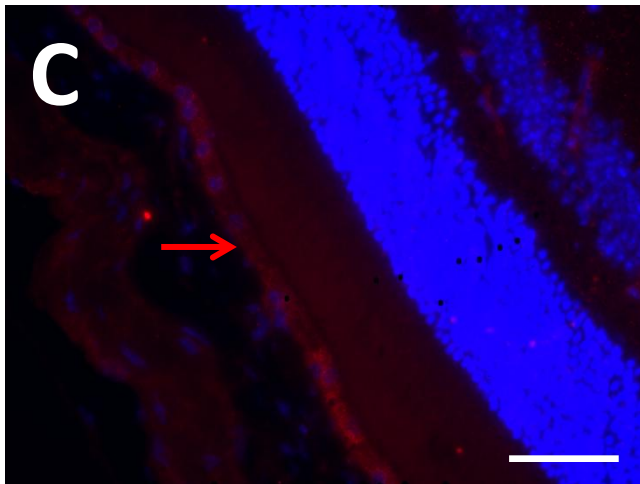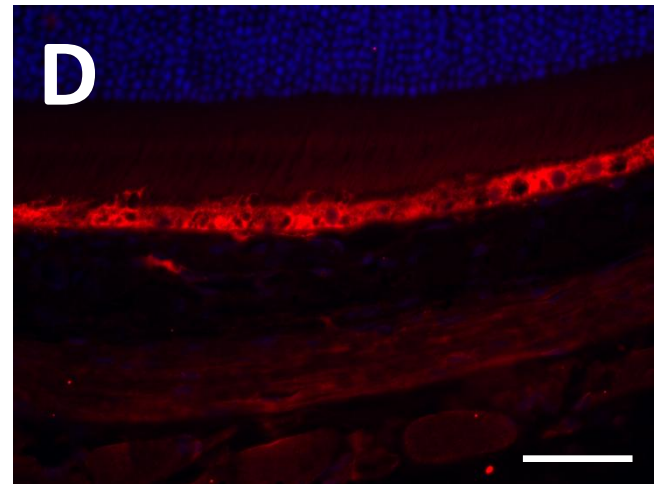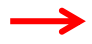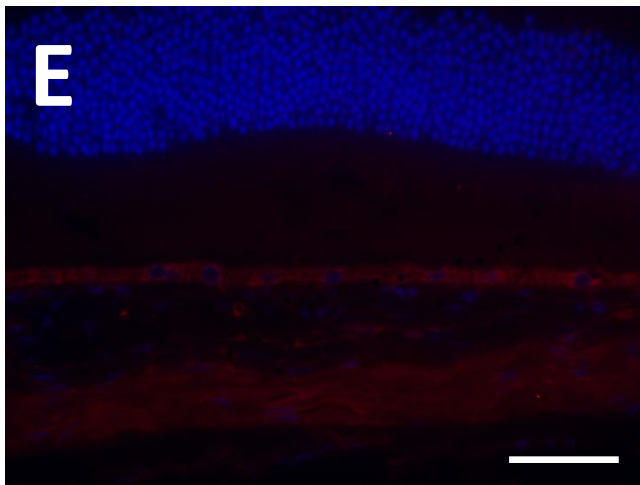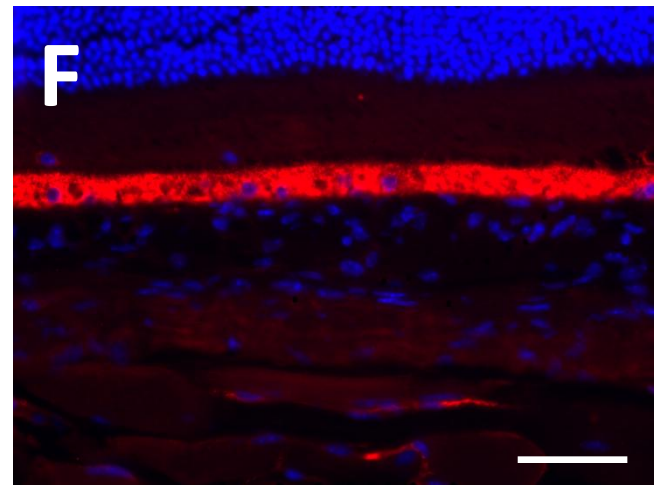

isotype control

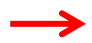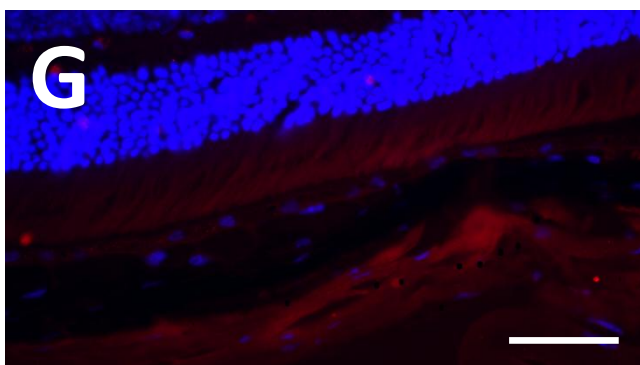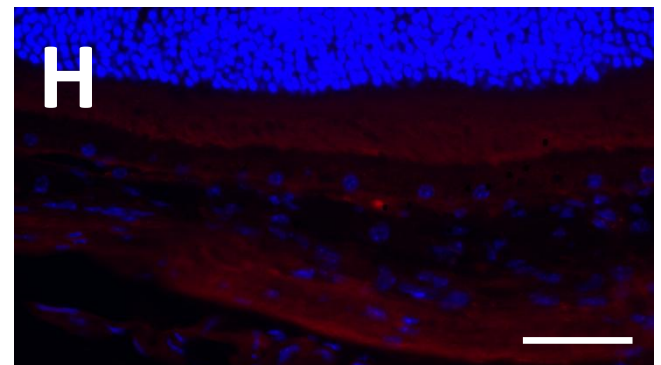

Supplement: S3 Fig — Immunofluorescence (IF) labelling of RPE65 (red) in Atg7f/f (A, C, E) and Atg7f/f Tyr-Cre (B, D, F) eyes. The mice were 10–12 months old. The results of 3 samples per genotype are shown (representative for >5 mice per genotype). Replacement of the anti-RPE65 antibody with an isotype control antibody abolished the specific labeling (G, H). Nuclear DNA was labelled with Hoechst 33258 (blue). The position of the RPE is indicated by a red arrow. Scale bars: 50 μm. (PDF) [file pone.0161640.s003.pdf]

*WT*

*Tyr-Cre*

**A**

**B**

DNA RPE65

DNA RPE65

**C**

**D**

DNA RPE65

DNA RPE65

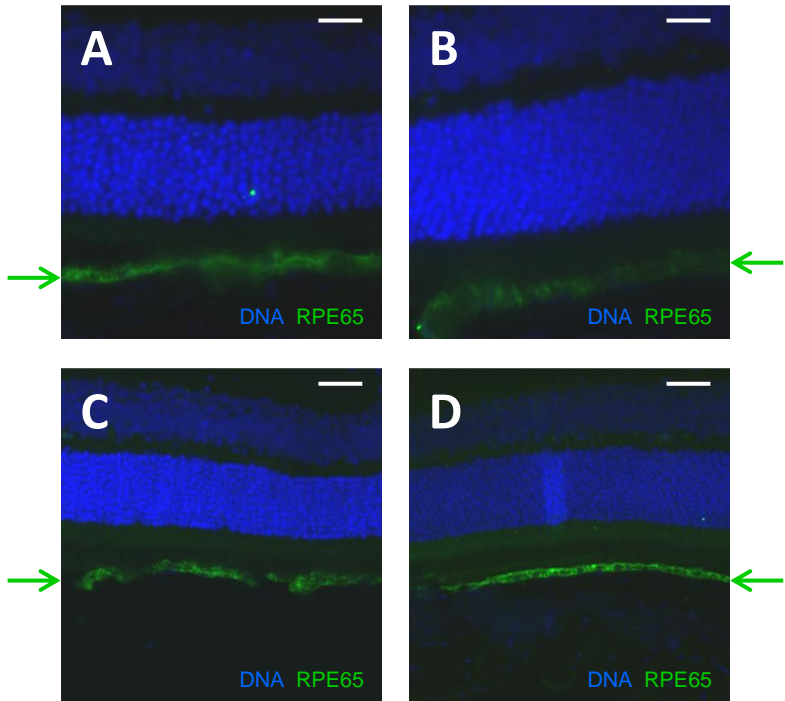

Supplement: S4 Fig — As a control experiment, sections through the eyes of wildtype (A, C) and Tyr-Cre (B, D) mice on a C57BL/6 background carrying the normal (not floxed) Atg7 gene were immunofluorescence labelled for RPE65 (green). Nuclear DNA was labelled with Hoechst 33258 (blue). The results are representative for 3 mice per genotype. The position of the RPE is indicated by a green arrow. Scale bars: 20 μm. (PDF) [file pone.0161640.s004.pdf]

**A**

*Atg7<sup>f/f</sup>*  
(C57BL/6)

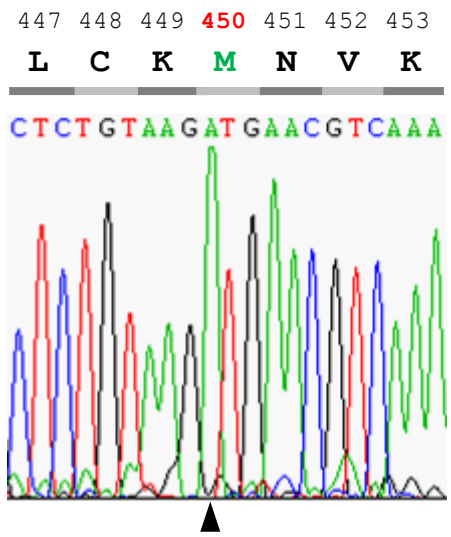**B**

*Atg7<sup>f/f</sup> Tyr-Cre*  
(C57BL/6)

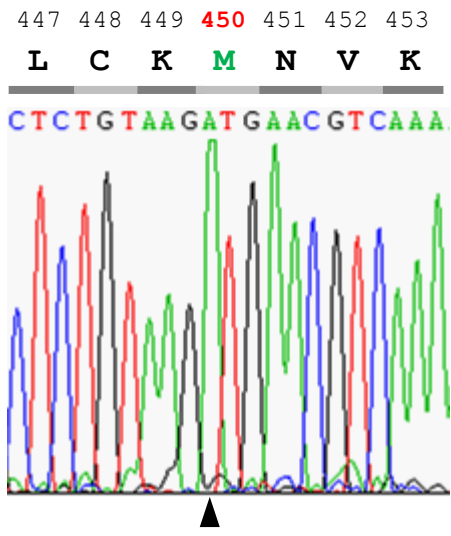**C**

BALB/c x C57BL/6

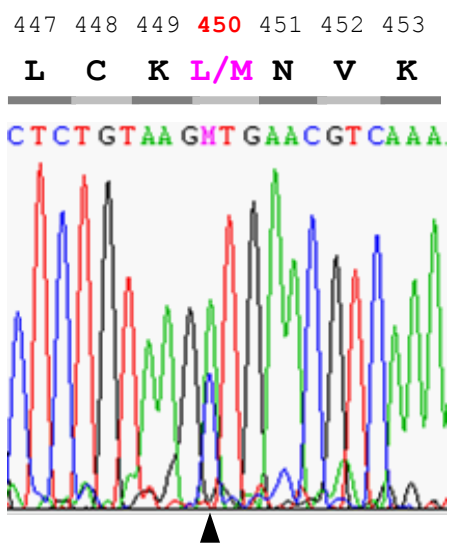

Supplement: S5 Fig — Genomic DNA was prepared from Atg7f/f (A) and Atg7f/f Tyr-Cre (B) mice from the same breeding colony. DNA from an unrelated mouse on a mixed BALB/c and C57BL/6 background (C) was investigated for comparison. The DNAs were amplified with Rpe65-specific primers as described in the Material and Methods section, and sequenced. Representative sequence chromatograms are shown. The positions of the single nucleotide polymorphism leading to either M450 (codon: ATG) or L450 (codon: CTG) are indicated by arrowheads. The encoded amino acid residues and the numbers of the residues in the RPE65 protein are shown above the chromatograms. (PDF) [file pone.0161640.s005.pdf]

# *Atg7<sup>f/f</sup>*

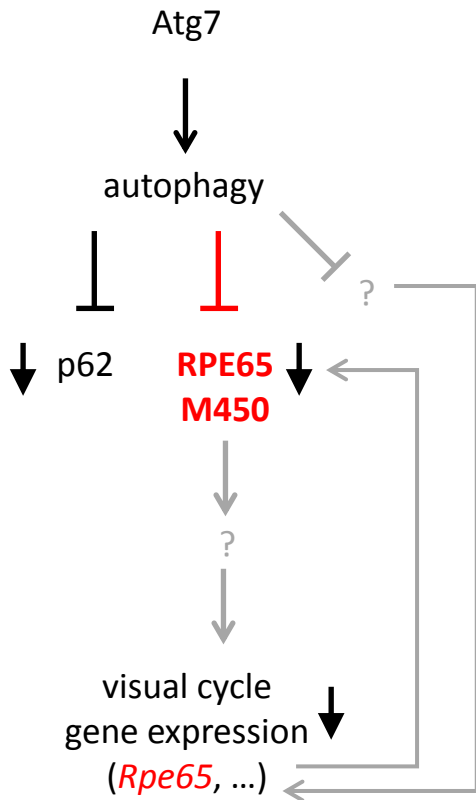

# *Atg7<sup>f/f</sup> Tyr-Cre*

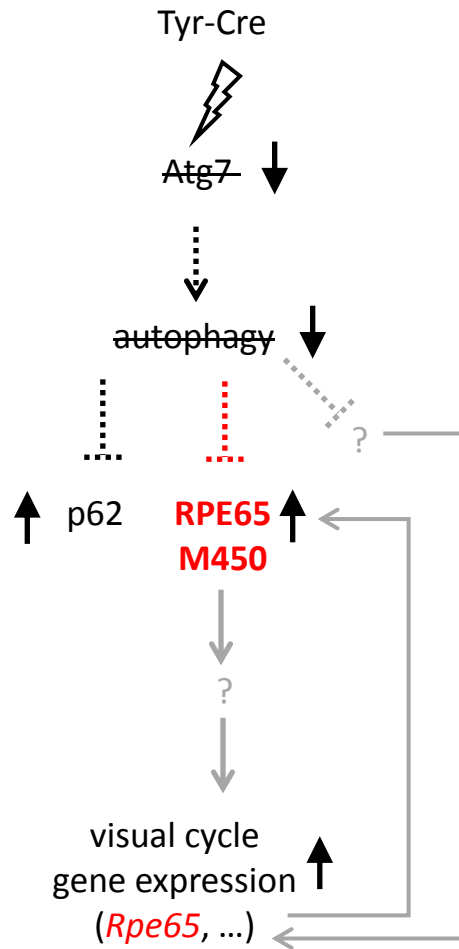

Supplement: S6 Fig — This model includes speculative elements besides changes observed in the study of Atg7f/f and Atg7f/f Tyr-Cre mice. According to this model, RPE65 M450 is largely degraded by autohpagy in RPE cells expressing Atg7 (Atg7f/f (left panel)). It is possible that autophagy influences the expression of visual cycle genes (Rpe65, Lrat, Rgr) via unknown mechanisms (indicated by question marks). In Atg7f/f Tyr-Cre RPE cells (right panel), RPE65 M450 is not degraded and reaches higher abundance. Possibly, the effect of RPE65 M450 on the retinoid metabolism or other mechanisms contribute to an upregulation of the transcripion of visual cycle genes including Rpe65. Importantly, several hypotheses depicted in this schematic remain to be tested in future studies. (PDF) [file pone.0161640.s006.pdf]
