# Supplementary material for: Tyrosinase-Cre-Mediated Deletion of the Autophagy Gene Atg7 Leads to Accumulation of the RPE65 Variant M450 in the Retinal Pigment Epithelium of C57BL/6 Mice
Source: PLoS One. 2016 Aug 18;11(8):e0161640. doi: 10.1371/journal.pone.0161640 (PMC4990303; doi:10.1371/journal.pone.0161640)
Supplement: S1 Table — (PDF) [file pone.0161640.s007.pdf]

Supplementary Table S1. Primers for quantitative RT-PCRs using LightCycler technology

Target gene	Sense primer	Antisense primer
Alas1	CCACTGGAAGAGCTGTGTGA	TGGCAATGTATCCTCCAACA
B2m	ATTCACCCCCACTGAGACTG	TGCTATTTCTTTCTGCGTGC
Lrat	ACTGCAGATATGGCTCTCGG	ACAGATTGCAGGAAGGGTCA
Otx2	ACTTCGGGTATGGACTTGCT	CCCTGGATTCTGGCAAGTTG
Rdh5	GGGCTACTGTGTCTCCAAGT	CGTAGTGGGCCTGTATAGCT
Rgr	TCGTACCCCTGTTCATCACA	GTGCAACGCATAGTTGATGG
Rpe65	TCTCTGTTGCTGGAAAGGGT	TTGTATGGGGCAGTGTGACT
Sox9	AGGAAGCTGGCAGACCAGTA	CGTTCTTCACCGACTTCCTC

Note: Primer sequences are shown in 5´-3´ orientation. Quantitative real-time PCR was performed using the LightCycler technology (Roche Applied Science, Mannheim, Germany) according to published protocols (Eckhart et al. Histidase expression in human epidermal keratinocytes: regulation by differentiation status and all-trans retinoic acid. J Dermatol Sci. 2008;50:209-15; Gruber et al. NF-E2-related factor 2 regulates the stress response to UVA-1-oxidized phospholipids in skin cells. FASEB J. 2010;24:39–48).
