# Supplementary material for: Tyrosinase-Cre-Mediated Deletion of the Autophagy Gene Atg7 Leads to Accumulation of the RPE65 Variant M450 in the Retinal Pigment Epithelium of C57BL/6 Mice
Source: PLoS One. 2016 Aug 18;11(8):e0161640. doi: 10.1371/journal.pone.0161640 (PMC4990303; doi:10.1371/journal.pone.0161640)
Supplement: S2 Table — (PDF) [file pone.0161640.s008.pdf]

S2 Table. Comparison of RPE65 amino acid (aa) sequences in vertebrates

Species	Species name	Accession number	RPE65 aa sequence (# 440-460)
Human	*Homo sapiens*	NP_000320.1	NHFVPDRLCKLNVKTKETWVW
Macaque	*Macaca mulatta*	XP_001095946.1	NHFVPDRLCKLNVKTKETWVW
**Mouse C57BL/6**	*Mus musculus*	NP_084263.2	NHFVPDKLCKMNVKTKEIWMW
Mouse BALB/c	*Mus musculus*	n.a.	NHFVPDKLCKLNVKTKEIWMW
Rat	*Rattus norvegicus*	NP_446014.2	NHFVPDKLCKLNVKTKEIWMW
Dog	*Canis lupus*	NP_001003176.1	NHFVPDRLCKLNVKTKETWVW
Cattle	*Bos taurus*	NP_776878.1	NHFVPDRLCKLNVKTKETWVW
Elephant	*Loxodonta africana*	XP_003411286.1	NHFVPDRLCKLNVKTKETWVW
Opossum	*Monodelphis domestica*	XP_007480467.1	NHFVPDRLCKLNVVTKETWLW
Platypus	*Ornithorhynchus anatinus*	XP_007671428.1	NHFVPDRLCKLNVKTKDTWVW
Alligator	*Alligator mississippiensis*	XP_006273895.1	NHFVPDRLCKLNIKTRETWVW
Chicken	*Gallus gallus*	NP_990215.1	NHFVPDRLCKLNVKTKETWVW
Ostrich	*Struthio camelus australis*	XP_009687334.1	NHFVPDRLCKLNVKTRETWVW
Chinese softshell turtle	*Pelodiscus sinensis*	XP_006123418.1	NHFVPDRLCKMNVKTKETWVW
Painted turtle	*Chrysemys picta bellii*	XP_005300179.1	NHFVPDRLCKLNVKTKETWVW
Gecko	*Gekko japonicus*	XP_015271276.1	NHFVPDRLCKMNVKTKETWVW
Green anole lizard	*Anolis carolinensis*	XP_003225885.1	NHFVPDRLCKINIKTRETWVW
Python	*Python bivittatus*	XP_007433640.1	NHFVPDRLCKMNVKTRETWVW
Frog	*Xenopus tropicalis*	NP_001120538.1	NHFIPDRLTKLNVKTKETWVW
Salamander	*Ambystoma tigrinum*	Q9YI25.3	NHFVPDRLSKLNVKTKETWVW
Coelacanth	*Latimeria chalumnae*	XP_006011673.1	NHFVPDRICKLNVKTKATWVW
Zebrafish	*Danio rerio*	NP_957045.1	NHFVPDRICKLNVRTKETWVW

Notes: Accession numbers refer to amino acid sequences deposited in GenBank. The sequence of RPE65 of the mouse BALB/c strain has been reported by Danciger et al. (2000). L450 of human RPE65 and the corresponding amino acid residues of other species´ RPE65 are highlighted. Note that the gecko, the green anole lizard and the python belong to the same phylogenetic clade, i.e. Squamata. aa, amino acid; n.a., not available.
